# Effect of CBCT voxel size and software program on the detection of fenestrations and dehiscences

**DOI:** 10.1186/s12903-026-07793-x

**Published:** 2026-02-20

**Authors:** Esraa Ahmed Eid, Walaa Mohamed Hamed, Mona Mahmoud Abo El Fotouh

**Affiliations:** Department of Oral and Maxillofacial Radiology, Faculty of Dentistry, Ain Shams University, Cairo, Egypt

**Keywords:** CBCT, Voxel size, Imaging software, Fenestration, Dehiscence, Alveolar bone defects

## Abstract

**Objectives:**

This study investigated the effect of CBCT voxel size and software program on the accuracy of detecting fenestrations and dehiscences.

**Materials and methods:**

This ex-vivo experimental animal study was carried out on 6 fleshy sheep heads with both maxilla and mandible accompanied with the surrounding soft tissue. Fenestrations [*n* = 48] and dehiscences [*n* = 51] were randomly created on the buccal surfaces of teeth. Two CBCT scans were acquired for each sheep head using two different voxel sizes [0.2 mm and 0.4 mm]. Images were analyzed by two oral radiologists on two different software programs [OnDemand and ImplaStation]. All data was collected and analyzed in terms of diagnostic performance measures.

**Results:**

For fenestrations, both voxel sizes and software programs showed high diagnostic performance, with approximately 94% accuracy and sensitivity, 95% specificity, and a low false positive rate of about 5%. For dehiscences, performance was more limited, with around 70% accuracy and sensitivity, 60–67% specificity, and a higher false positive rate of 33–40%. None of the investigated parameters showed statistically significant differences.

**Conclusion:**

Changes in voxel size and software program had little impact on the diagnostic accuracy for detecting fenestrations and dehiscences. CBCT demonstrated good performance in identifying fenestrations but only moderate capability in detecting dehiscences. These results indicate that, although CBCT is a useful imaging tool, its contribution to diagnosing dehiscences is limited by factors such as volume averaging and partial volume effects, and should therefore be interpreted with caution in clinical settings.

**Clinical relevance:**

Clinically, the findings suggest that choosing between 0.2 mm and 0.4 mm voxel sizes, or between different viewing software programs OnDemand and ImplaStation, does not meaningfully affect the ability to diagnose fenestrations and dehiscences. This provides flexibility in routine practice, allowing clinicians to select imaging settings based on practical considerations such as field of view, dose, or workflow, without compromising diagnostic confidence.

## Introduction

Fenestrations and dehiscences are periodontal defects that generally occur in the natural dentition. Fenestration is a localized ‘window-like’ defect in the cortical bone where the root surface is exposed but the marginal alveolar bone remains intact. While dehiscence is a cortical bone defect in which the root surface is exposed along with loss of the marginal alveolar bone [1]. Both defects are depicted together in Fig. 3. Some of the causative factors of these defects are clenching, bruxism extensive root curvature, labial tooth protrusion, traumatic occlusion, and tooth movement along with the thin cortical plate [2].

Fenestrations and dehiscences can significantly affect the outcomes of orthodontic, periodontal, and endodontic treatments, and may aggravate associated complications [3]. Marginal bone defects can influence the success of endodontic surgeries, with optimal outcomes occurring when buccal bone thickness exceeds 3 mm [4]. In implant dentistry, insufficient cortical support increases the risk of complications, and long-term success is more predictable when the alveolar bone is at least 2 mm thick [5]. These defects can also complicate implant placement by reducing primary stability and increasing the likelihood of dehiscence or implant exposure [6]. For orthodontic cases particularly labially positioned teeth, assessing alveolar bone for fenestration or dehiscence is essential, as rapid tooth movement can intensify bone loss [7].

Although clinical methods like periodontal probing and sounding are effective in evaluating pocket depth and attachment loss, imaging is necessary to determine the nature and extent of alveolar bone defects [8]. The use of two‑dimensional [2D] imaging modalities, as depicted in periapical, bitewing and panoramic radiographs, in the diagnosis of this type of periodontal defect is limited owing to the 2D nature and overlapping of the anatomical structures [9, 10]. However, CBCT can provide accurate analysis of periodontal defects due to lack of superimposition, 1:1 measurement, absence of geometric distortions, and three-dimensional [3D] display [11, 12].

Several studies have explored the efficacy of both 2D and 3D imaging modalities for diagnosing periodontal defects. Some of these studies demonstrated the accuracy of CBCT in evaluating periodontal defects in comparison validated clinical or surgical references [13–18]. Other studies reported the superiority of CBCT over 2D imaging when detecting dehiscences, fenestrations, furcation involvement, three-wall defects, and infrabony defects [19–23]. Despite its superiority, CBCT radiation exposure must be carefully managed in compliance with the ALADA principle [24].

The accuracy of CBCT is affected by several factors, such as voxel size, exposure settings, field of view, and image manipulation software. Voxel size is considered a critical determinant of CBCT image quality, where smaller voxel sizes provide higher spatial resolution and better diagnostic accuracy of periodontal bone defects [25, 26]. However, higher resolution is associated with higher radiation dose, more image noise, and larger file sizes. Accordingly, the choice of voxel size should seek to balance diagnostic reliability with patient safety [24, 27–29] .

Several studies have explored the influence of CBCT voxel size on the ability to detect fenestrations and dehiscences, but the results remain ambiguous. Some studies report that small differences in voxel sizes such as between 0.125 mm and 0.2 mm may not considerably affect diagnostic accuracy, whereas larger differences like between 0.2 mm and 0.4 mm could lead to apparent changes in detection rates [30]. Other studies documented that smaller voxel sizes are more effective in identifying minor defects, while showing no added value for larger defects [31–34]. On the contrary, A previous study reported that a larger voxel size [0.160 mm] showed higher accuracy than a smaller one [0.125 mm] [35].

Our study was designed to help address this conflict in literature and assist in creating standardized imaging protocols for detecting these specific periodontal defects. To this end, we focused on two commonly used CBCT voxel sizes, 0.2 mm and 0.4 mm, as they are commonly used in clinical CBCT imaging and represent a balance between image quality and radiation dose [30–32, 36, 37].

In addition to voxel size, the CBCT software plays a vital role in determining diagnostic accuracy. Different software programs vary in their tools for image rendering, contrast adjustment, brightness control, measurement accuracy, and 3D rendering features. Fundamental features such as multiplanar reconstruction [MPR], zoom and rotation controls, and linear measurement tools, and 3D volume rendering directly affect the detection of fine defects as fenestrations and dehiscence [28]. Additionally, many current software programs incorporate AI-based image reconstruction, which can help minimize artifacts and further improve diagnostic clarity [38].

Many studies have compared the performance of different CBCT software programs across different dental applications [39–43]. However, to the best of our knowledge, there is a clear scarcity of studies specifically comparing the performance of different CBCT software programs in detecting fenestrations and dehiscences. This research gap motivated the present study to explore this under-investigated area and contribute to a more evidence-based approach in diagnostic imaging.

## Materials and methods

### Study design and ethics approval

The current study was an ex-vivo experimental animal study that was carried out on 6 fleshy sheep heads with both maxilla and mandible accompanied with the surrounding soft tissue. The sheep heads used in this study were obtained from a local butcher shop after the animals had been slaughtered for human consumption. No animals were slaughtered specifically for research purposes. As these specimens represent by-products that would otherwise be discarded, informed consent from the butcher or owners was not applicable. The study did not involve any live animals, animal experimentation, or euthanasia procedures. This research received an ethics exemption from the Ethics Committee of the Faculty of Dentistry, Ain Shams University, as it does not involve live animals or human participants.

### Power analysis & sample selection

To compare the specificity between two different voxel sizes [0.2 and 0.4], it was estimated that a minimum of 58 teeth will be needed using on Beam’s method for comparing matched diagnostic tests [44]. The expected specificity of the 0.2 and 0.4 voxel sizes was based on the findings of a previous study [30] [77% and 92% respectively]. A significance level of 5% and power of 80% were assumed. The sample included a total of 144 teeth. Each of the six sheep heads provided three premolars and three molars from every quadrant. The selection criteria required that the skulls have an intact dentoalveolar structure with no signs of previous trauma and a complete set of posterior teeth.

### Defects creation

**Fig. 1:**
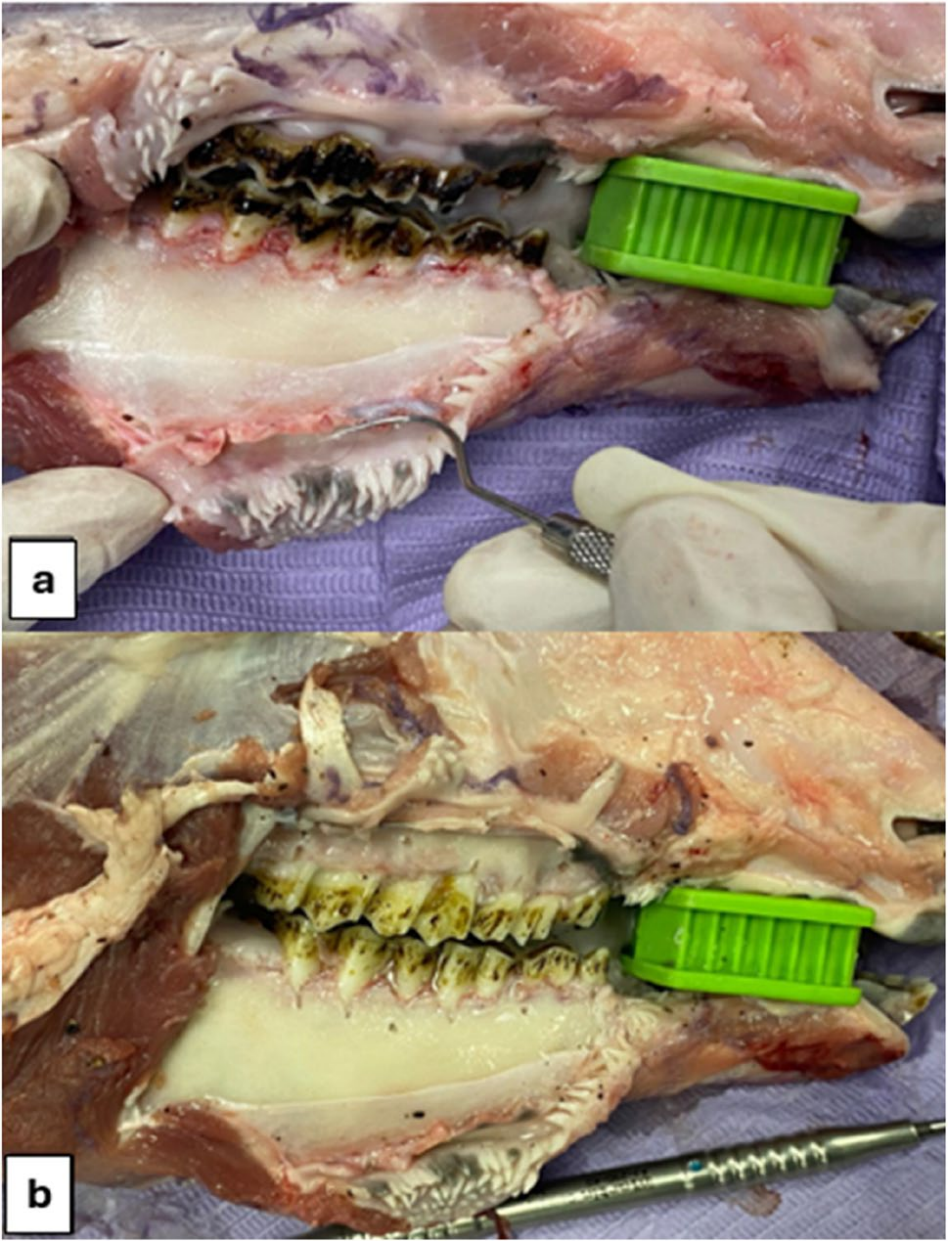
Clinical image of the sheep head after flap retraction in the right mandibular premolar-molar area. (**A**) before and (**B**) after scaling, root planning of the teeth and creation of the bony defects

Six flayed sheep heads with both maxilla and mandible attached normally at the temporomandibular joints accompanied with the surrounding flesh were provided. A mucoperiosteal flap was created using #15 surgical blade at each quadrant extending from the first premolar to the third molar. A mucoperiosteal elevator was used to raise the flap and clearly expose the underlying bone. The teeth in both jaws underwent scaling and root planning by hand instruments with precise adherence to the infection control measures (Fig. 1).

Next, 48 fenestrations and 51 dehiscences were randomly created across the teeth, with each tooth receiving either a fenestration or dehiscence. All defects were prepared by a single operator using 8‑fluted round dental carbide burs [size ¼, 0.5 mm diameter at the widest point] in a high‑speed handpiece under water coolant and direct visualization, following standardized dimensions and locations. The presence, type, and extent of each defect were documented at the time of preparation and served as the reference [gold] standard for subsequent image evaluation. A periodontal probe was used to keep the dimensions of the fenestration not more than 1.5 × 1.5 [length x width] and that of the dehiscence at least 2 mm length but not more than 3 × 1.5 [length x width]. Both types of defects were created at the buccal surface only where fenestrations were mostly at the middle third of the roots of both premolar and molar teeth, while dehiscences were created at the coronal third of the roots of premolars only (Fig. 1).

After defects were created, the key was written, and photographic pictures were taken. Next, flaps were returned to their primary position and fixed with 2 simple sutures using non-absorbable suture thread [3/0 USP] at the 2 ends of the flap.

### CBCT imaging acquisition and analysis

CBCT scans for sheep heads were acquired twice using Planmeca ProMax^®^ 3D Mid CBCT scanner [Planmeca, Helsinki, Finland] using two different voxel sizes 0.2 and 0.4, operating at tube voltage 90 kVp, tube current 12.5 mA, field of view 20 × 10 cm, and scanning time 18 s. To be observed, DICOM files of both 0.2 and 0.4 voxel sizes CBCT scans were imported to OnDemand 3D App software [version 1.0.0.1 provided by Cybermed, Seoul, Korea. To compare the two viewing software programs, only the 0.2 mm voxel datasets were imported into ImplaStation [version 4.6.0. provided by ProDigiDent, USA]. This choice was made to isolate the effect of the software by keeping all acquisition parameters, including voxel size, constant between OnDemand and ImplaStation, while the influence of voxel size [0.2 mm vs. 0.4 mm] was evaluated separately within OnDemand.

**Fig. 2:**
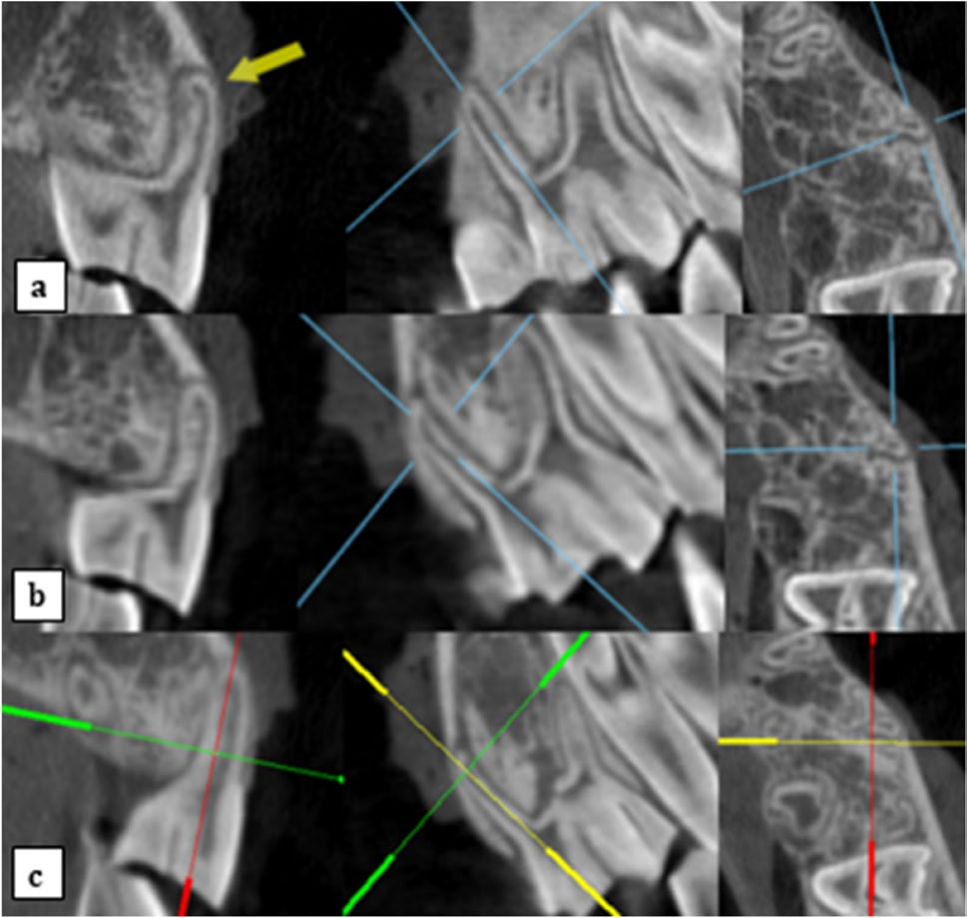
Oblique coronal sections, accompanied by their corresponding sagittal and axial reconstructions, illustrating fenestration on a premolar root displayed using OnDemand software at voxel sizes of 0.2 mm (**a**) and 0.4 mm (**b**), and the ImplaStation software at a voxel size of 0.2 mm (**c**)

Images were observed twice by two oral radiologists, with a minimum of five years of experience in CBCT images interpretation with one-month time interval between both observations. Scans were coded by the main researcher in this study who was not involved in the observations. Readings were observed in a random sequence, and the observers were blinded. To decrease bias, both observers agreed on a certain protocol for observation. Images were observed on the corrected coronal cuts of the MPR screen of both CBCT software programs, where the line representing the sagittal cut was aligned parallel to the long axis of the buccal root of the involved tooth and accordingly, the line representing the axial cut was aligned perpendicular to this line (Fig. 2).

**Fig. 3:**
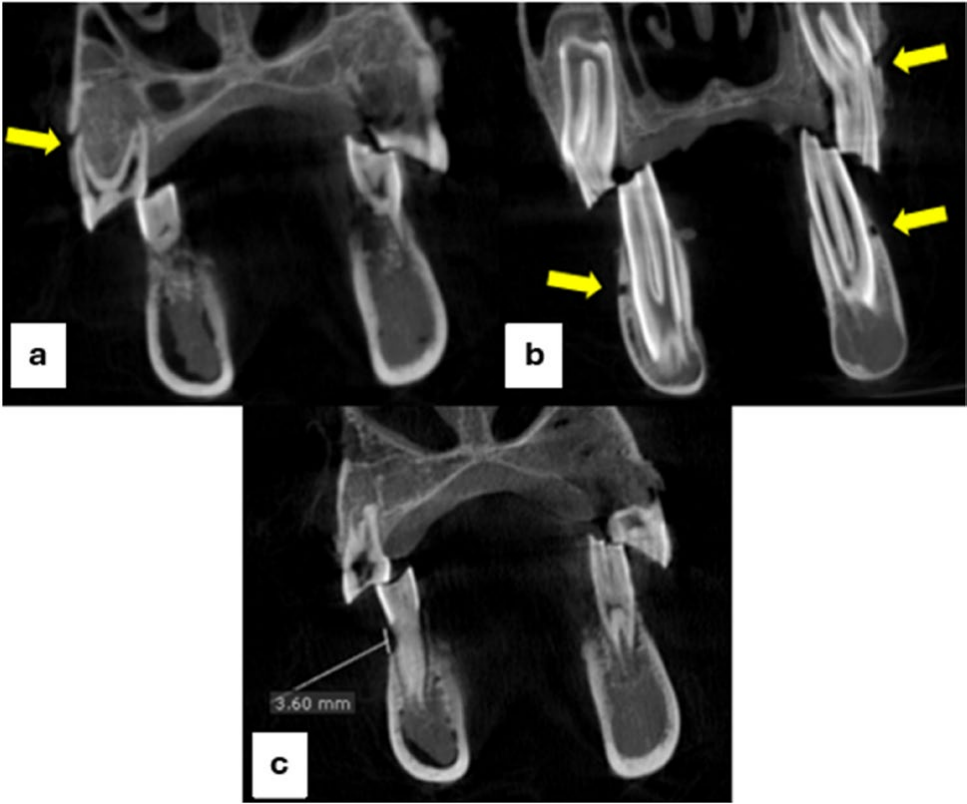
Corrected coronal cut of OnDemand CBCT software at a voxel size of 0.2 mm showing: (**A**) a fenestration on the buccal root of the upper right 2nd premolar [arrow], (**B**) fenestrations on the buccal roots of the upper left, lower right and left 1st molars [arrows], and (**C**) a dehiscence on the buccal root of the lower right 2nd premolar with 3.6 mm bone loss from the alveolar crest

Fenestration was traced along the length of the root, while dehiscence was considered only when the bone loss from the alveolar crest exceeds 2 mm using the linear measuring tool in both software programs in the premolar teeth (Fig. 3). All readings were collected by the main researcher in the form of tables sent for statistical analysis.

### Statistical analysis

All observers’ readings for fenestration and dehiscence were compared with the clinical key in terms of accuracy, sensitivity, specificity, false positive rate [FPR], positive predicted values [PPV], and negative predicted values [NPV] for the OnDemand program using 0.2 mm, 0.4 mm voxel sizes and ImplaStation program using 0.2 mm voxel size. Descriptive statistics were calculated for each parameter separately and the mean were used for graphical visualization.

Cumulated values of both observers for fenestration and dehiscence separately were tested for normality by Shapiro wilk test, compared using Kruskal-Wallis Test followed by pairwise Mann-Whitney test since most of the data was non-parametric. Bonferroni equations were used for adjustment. Alpha value used was 0.05. For comparing between the fenestration and dehiscence results at all previously mentioned parameters ANOVA two factors were used with alpha value also 0.05. Inter-rater and intra-rater reliability were evaluated using the intraclass correlation coefficient [ICC] and interpreted according to the classification proposed by Regier et al.

## Results

### Intra and Inter-observer reliability

**Fig. 4:**
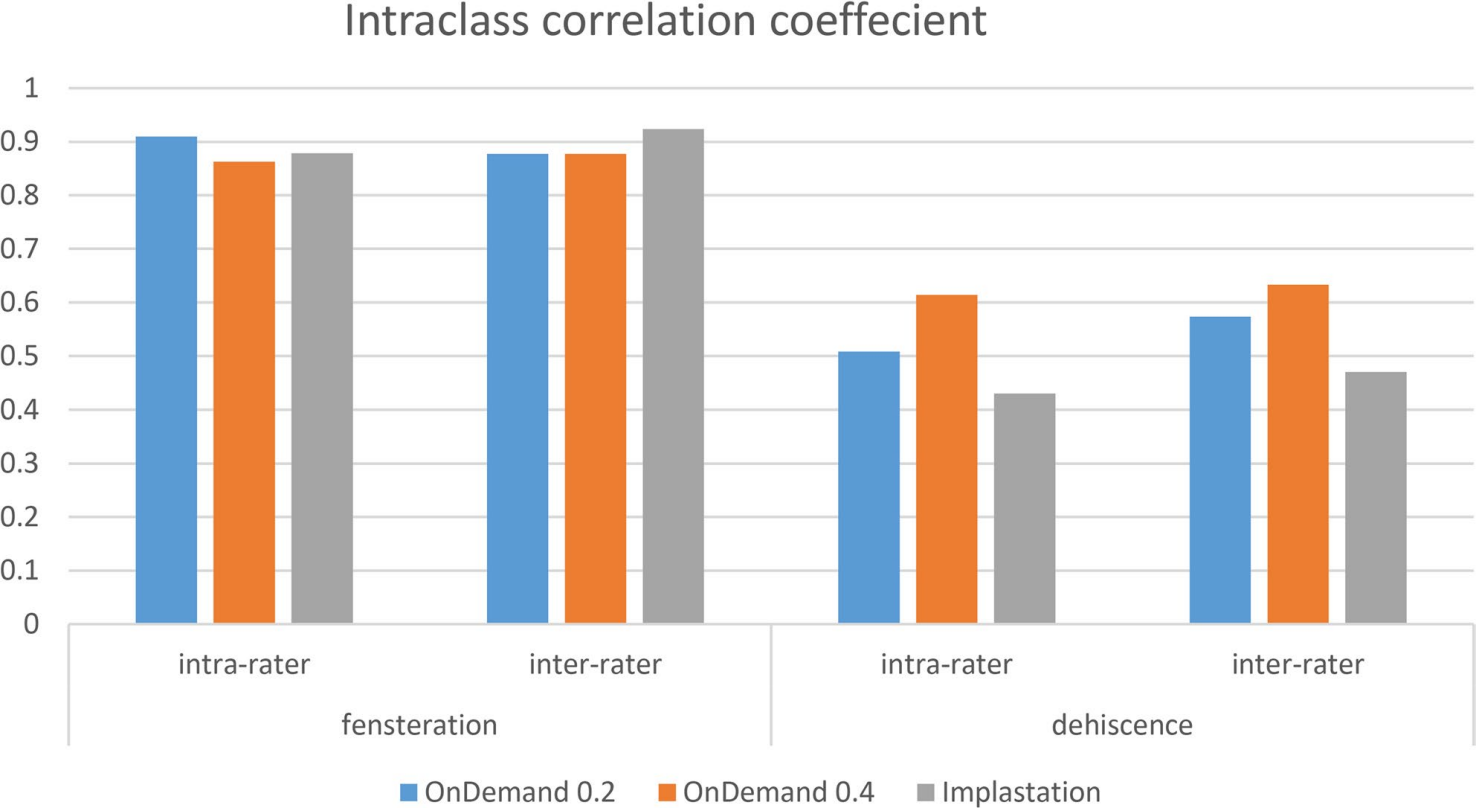
A bar-graph showing intra and inter-rater results of both fenestrations and dehiscences upon the readings of both voxel sizes and software programs

In the assessment of fenestration, both intra-observer and inter-observer reliability were excellent across all variables studied, including OnDemand with 0.2 mm voxel size, OnDemand with 0.4 mm voxel size, and Implastation with 0.2 mm voxel size. For dehiscence, the reliability was relatively lower, showing good agreement between and within observers for all tested variables (Fig. 4).

### Effect of CBCT voxel size on the detection of fenestrations and dehiscences

The mean values of the diagnostic accuracy measures for OnDemand at both 0.2 mm and 0.4 mm voxel sizes are presented in Table 1, covering both fenestration and dehiscence assessments. For fenestrations, both voxel sizes demonstrated excellent performance in terms of accuracy, sensitivity, specificity, PPV, and NPV, along with an exceptionally low FPR. While for dehiscences, the diagnostic measures remained very good overall, though the FPR was found to be less consistent and somewhat questionable. Mann–Whitney U test revealed no statistically significant differences between the 0.2 mm and 0.4 mm voxel sizes in the OnDemand software for either type of defect, with all p-values exceeding the alpha level of 0.05 (Table 1).

### Effect of CBCT software program on the detection of fenestrations and dehiscences

The mean values of the diagnostic accuracy measures for both OnDemand and ImplaStation CBCT software were calculated and are presented in Table 2 for both fenestrations and dehiscences. For fenestration detection, both programs demonstrated excellent levels of accuracy, sensitivity, specificity, PPV and NPV, along with a very low FPR. When it came to dehiscence detection, both software programs still showed strong performance across all measures. Mann–Whitney U test no statistically significant differences between the OnDemand and ImplaStation software programs for either type of defect, with all *p*-values exceeding the alpha level of 0.05 (Table 2).

**Table 1:** Mean values of diagnostic accuracy measures for fenestration and dehiscence detection in 0.2 mm and 0.4 mm voxel sizes

Parameter Mean	Accuracy	Sensitivity	Specificity	FPR	PPV	NPV
Fenestrations						
OnDemand 0.2	94.5 [91–97]	93.8 [88–97]	94.8 [91–97]	5.2 [3–9]	90.2 [84–95]	96.8 [93–99]
OnDemand 0.4	95.0 [91–97]	93.8 [88–97]	95.6 [92–98]	4.4 [2–8]	91.5 [85–96]	96.9 [93–99]
*P*-value	0.56	1.00	0.56	0.56	0.47	0.89
Dehiscences						
OnDemand 0.2	71.2 [61–81]	71.6 [59–85]	66.8 [50–77]	33.2 [23–50]	76.2 [62–85]	62.9 [48–76]
OnDemand 0.4	69.3 [62–78]	71.7 [60–85]	61.1 [39–75]	38.9 [25–61]	71.9 [60–83]	61.1 [45–74]
*P*-value	0.77	1.00	0.43	0.47	0.79	0.77

Statistical significance assessed using Mann–Whitney U test; *p* < 0.05 considered significant. Values are expressed as percentage mean [95% confidence interval]

**Table 2:** Mean values of diagnostic accuracy measures for fenestration and dehiscence detection in ondemand and implastation CBCT software programs

Parameter Mean	Accuracy	Sensitivity	Specificity	FPR	PPV	NPV
Fenestrations						
OnDemand	94.5 [91–97]	93.8 [88–97]	94.8 [91–97]	5.2 [3–9]	90.2 [84–95]	96.8 [93–99]
ImplaStation	95.0 [91–97]	94.3 [89–97]	95.3 [92–98]	4.7 [2–8]	91.0 [85–95]	97.1 [94–99]
*P*-value	0.77	0.76	0.66	0.66	0.89	0.88
Dehiscences						
OnDemand	71.2 [61–81]	71.6 [59–85]	66.8 [50–77]	33.2 [23–50]	76.2 [62–85]	62.9 [48–76]
ImplaStation	68.8 [60–77]	73.5 [66–81]	59.9 [43–75]	40.1 [25–57]	72.5 [63–81]	60.9 [47–74]
*P*-value	0.66	0.88	0.66	0.38	0.38	0.66

Statistical significance assessed using Mann–Whitney U test; *p* < 0.05 considered significant. Values are expressed as percentage mean [95% confidence interval]

### Comparison between both CBCT Factors; voxel size and software program

Kruskal-Wallis test was used to determine whether there were significant differences between the cumulative values of the raters’ observations across the three readings [OnDemand 0.2, OnDemand 0.4, and ImplaStation] for fenestrations and dehiscences. The test revealed *no statistically significant differences*, as the p-values were greater than the assigned alpha level [0.05], with 95% confidence intervals observed across all parameters for both fenestrations and dehiscences regardless of the software program or voxel size (Table 3).

**Table 3:** *p*- values of diagnostic accuracy measures for fenestration and dehiscence detection for both voxel sizes and software programs assessed using Kruskal-Wallis test

Parameter	Accuracy	Sensitivity	Specificity	FPR	PPV	NPV
Fenestration	0.79	0.9	0.73	0.73	0.73	1.00
Dehiscence	0.75	0.97	0.51	0.51	0.08	0.83

### Comparison between fenestration and dehiscence results

To compare the diagnostic accuracy measures for both fenestration and dehiscence, a two-way ANOVA test was conducted using an alpha level of 0.05. Despite the higher diagnostic accuracy measures for fenestrations than dehiscences, no statistically significant differences were found between the three software settings: OnDemand with 0.2 mm voxel size, OnDemand with 0.4 mm voxel size, and ImplaStation with 0.2 mm voxel size (Table 4).

**Table 4:** Comparison between fenestration and dehiscence results across the three readings

	OnDemand 0.2			OnDemand 0.4		Implastation	
	F	D	F		D	F	D
*Accuracy*	94.5	71.2	95.0		69.3	95.0	68.8
*Sensitivity*	93.8	71.6	93.8		71.7	94.3	73.5
*Specificity*	94.8	66.8	95.6		61.1	95.3	59.9
*FPR*	5.2	33.2	4.4		38.9	4.7	40.1
*PPV*	90.2	76.2	91.5		71.9	91.0	72.5
*NPV*	96.8	62.9	96.9		61.1	97.1	60.9
*p-value*	0.35		0.38			0.39	

*F* Fenestration and *D* Dehiscence

## Discussion

Accurate identification of fenestrations and dehiscences is essential as they may affect the treatment plan of endodontic surgeries, orthodontic treatments, implant placement and other dental procedures [3–7]. CBCT has proven to be a suitable diagnostic tool for evaluating this type of defect [19–23]. Accordingly, the current study was concerned with proposing the best imaging and observation protocol regarding the voxel size and manipulation software to decrease the probability of faulty diagnosis and enhance the outcome of these dental procedures.

Fleshy sheep heads were selected for better simulation of the natural soft tissue and its associated image noise in CBCT scans. Defects were created exclusively on the buccal surfaces as several studies have demonstrated that fenestrations and dehiscences occur more frequently on the buccal surfaces of teeth compared to the lingual surfaces [3, 7, 45–52]. Dehiscences were created in the cervical third of the alveolar ridge, as this has been reported to be their most common location in humans according to Nalbantoğlu et al. [53] and Kajan et al. [45]. In contrast, fenestrations were created in the middle third of the alveolar ridge, despite multiple studies [3, 7, 45, 48, 49, 53] indicating that they more commonly occur in the apical third. This deviation was due to the clinical difficulty in identifying the exact root apex. Since sheep molar teeth lack a CEJ, which is necessary for identifying this type of defect, dehiscences were created and assessed solely on premolar teeth where the CEJ is present.

The 0.2 mm voxel size was put under investigation in the current study since it is used as a default setting in many CBCT systems, providing a balance between high image quality and acceptable exposure dose and time [30, 32, 36, 37]. Alternatively, the 0.4 mm voxel size is typically employed for scans requiring a larger field of view, as it provides sufficient image quality while reducing radiation exposure and scan duration [30, 31, 36].

Regarding the selection of the two CBCT software programs, OnDemand was selected due to its broad use as a third-party application, where it is available under a commercial license [31, 34, 54, 55]. Concerning ImplaStation, it is a user-friendly and readily accessible CBCT software program that provides a wide range of functions free of charge. The involvement of this software was very limited in previous studies; this could be attributed to its relatively recent introduction to the market in 2016.

The results of the current study revealed excellent diagnostic performance of CBCT in identifying fenestrations and acceptable performance for dehiscences, with no significant differences between 0.2 mm and 0.4 mm voxel sizes or between OnDemand and ImplaStation software. Although smaller voxels offer higher resolution, they may increase image noise; in our study, voxel size had minimal impact on diagnostic accuracy and reliability. A comparison between the current study and other studies Investigating the Effect of CBCT Voxel Size on Diagnostic Accuracy of Alveolar Bone Defects is summarized in Table 5.

**Table 5:** A comparison between the current study and other studies investigating the effect of CBCT voxel size on diagnostic accuracy of alveolar bone defects

Study	Voxel Sizes Compared	Defects Assessed	Main Findings	Conclusion
The current study	0.2, 0.4 mm	Fenestrations, Dehiscences	No significant difference between 0.2 and 0.4 mm	Changes in voxel size have little effect on diagnostic accuracy.
Kolsuz et al. [32]	0.08, 0.1, 0.125, 0.15, 0.16, 0.2 mm	Fenestrations, Dehiscences	Smaller voxel sizes [≤ 0.125 mm] showed higher accuracy. 0.200 mm performed worst.	Voxel size ≤ 0.125 mm recommended for accurate detection.
Dong et al. [30]	0.125, 0.2, 0.4 mm	Fenestrations, Dehiscences	No significant difference between 0.125 and 0.2 mm. 0.4 mm had significantly lower accuracy.	Voxel sizes ≥ 0.4 mm reduce diagnostic accuracy.
Aasy et al. [31]	0.25, 0.4 mm	Fenestrations, Dehiscences, Infrabony defects	0.25 mm significantly better for small defects [1–2 mm]. No difference for larger defects.	Smaller voxels improve accuracy in small defects.
Eftekhar et al. [33]	0.15, 0.3 mm	Fenestrations, Dehiscences	0.15 mm superior for both defects; 0.30 mm reduced accuracy.	Smaller voxels improve accuracy, especially for fenestrations.
Foroozandeh et al. [34]	0.09, 0.18, 0.3 mm	Fenestrations, Dehiscences	0.18 mm best for dehiscences; 0.09 mm best for fenestrations.	Different voxel sizes optimal depending on defect type.
Hatata et al. [37]	0.1, 0.2, 0.25 mm	1-, 2-, 3-walled infrabony defects	0.25 mm had frequent errors, especially in BL and volumetric dimensions.	Voxel size 0.25 mm less suitable for fine structures.
De Oliveira et al. [36]	0.08–0.16 mm vs. 0.3–0.4 mm [across systems]	Alveolar bone levels [no defects]	Smaller voxels [0.08–0.16 mm] matched gold standard; larger voxels showed deviations.	Small voxels more reliable for bone level assessment.
Icen et al. [35]	0.125, 0.16 mm	Fenestrations, Dehiscences	0.160 mm outperformed 0.125 mm, possibly due to less image noise.	Slightly larger voxels may reduce noise and improve detection.
Van Leeuwen et al. [56]	0.2, 0.4, 0.6 mm	Fenestrations, Dehiscences	Fenestrations undetectable at all voxel sizes. Dehiscence overestimated with larger voxels.	CBCT clinically acceptable for dehiscences, not fenestrations

These findings differ from those of Kolsuz et al. [32], who investigated six voxel sizes ranging from 0.080 mm to 0.200 mm. They reported that smaller voxel sizes [≤ 0.125 mm] significantly outperformed larger ones in detecting fenestrations and dehiscences. Moreover, Dong et al. [30] examined the diagnostic reliability of 0.125 mm, 0.2 mm, and 0.4 mm voxel sizes. Unlike the current study, they reported that while 0.125 mm and 0.2 mm produced comparable results, the 0.4 mm group demonstrated significantly lower diagnostic efficacy.

Furthermore, Aasy et al. [31] demonstrated a significant difference between 0.25 mm and 0.4 mm voxel sizes for detecting smaller dehiscences [1–2 mm] and fenestrations [1–3 mm], with no significant differences observed for larger defects [≥ 4 mm]. In contrast, the current study did not account for defect size, which could explain the differing outcomes. Eftekhar et al. [33] evaluated the diagnostic performance of 0.15 mm and 0.30 mm voxel sizes. Although their conclusions concur with our findings that fenestrations are generally easier to detect than dehiscences, they reported a significant difference between voxel sizes.

A previous study conducted by Foroozandeh et al. [34] evaluated different acquisition settings using various voxel sizes and fields of view. Their findings demonstrated significantly higher diagnostic accuracy of smaller voxel sizes [e.g., 0.09 mm] in fenestrations detection, while dehiscences were best detected using medium voxel sizes [0.18 mm]. This variation from the results of the current study may be attributed to the use of much smaller voxel sizes used in their research.

Hatata et al. [37] assessed the diagnostic accuracy of three different voxel sizes of 0.1 mm, 0.2 mm, and 0.25 mm. They reported that 0.25 mm voxel size significantly resulted in measurement inaccuracies. However, our study did not observe this statistically significant difference between voxel sizes. This may be related to the different nature of defects evaluated; Hatata et al. investigated infrabony defects, which may present different imaging challenges compared to fenestrations and dehiscences.

De Oliveira et al. [36] demonstrated that smaller voxel sizes [e.g., 0.08–0.16 mm] produced results that were statistically indistinguishable from the gold standard, while larger voxel sizes [e.g., 0.3–0.4 mm] were significantly different. This differs from our results, where both 0.2 mm and 0.4 mm voxel sizes provided acceptable diagnostic reliability. Major differences from the current study that may be the cause of this deviation in the results is that unlike our study, De Oliveira et al. examined the combined effect of field of view and used larger voxel sizes overall.

In contrast to the results of our study and all previously mentioned ones, Icen et al. [35] reported unexpectedly better diagnostic accuracy with the larger voxel size [0.160 mm] in comparison to 0.125 mm voxel size. A possible explanation of this discrepancy is the extremely small voxel sizes they used. Van Leeuwen et al. [56] reported that fenestrations could not be detected at any voxel size in their study [0.2 mm, 0.4 mm, and 0.6 mm], while dehiscences can be accurately detected using smaller voxel sizes with a significant difference from the larger ones. These findings contrast with the current study, where fenestrations were more accurately detected than dehiscences, and no significant difference was demonstrated between 0.2 mm and 0.4 mm voxel sizes.

To the best of our knowledge, no prior studies have directly compared CBCT software programs for detecting fenestrations and dehiscences. A relatively close study was conducted by Hatata et al. [37] who compared the accuracy of OnDemand 3D and OsiriX in linear and volumetric measurements of periodontal defects. They reported no significant difference between the two software platforms. However, they observed significant differences in measurement accuracy when comparing voxel sizes within each software—specifically between 0.1 mm, 0.2 mm, and 0.25 mm. This indicates that while software selection may not impact overall accuracy, the combined effect of both voxel size and software can influence precision. These results align with our findings in demonstrating no significant difference between software programs in detecting alveolar bone defects.

A major strength of the current study is its dual focus on two crucial factors affecting the detection of fenestrations and dehiscences: voxel size used during image acquisition and the software used for image interpretation. Each was assessed separately, followed by a direct comparison to comprehend their individual and combined impact.

However, some limitations should be noted. Firstly, the anatomical differences of sheep heads may limit the direct applicability to humans. The absence of cementoenamel junction in molar teeth limited the ability to detect dehiscences in these sites. Additionally, the absence of metal restorations—commonly present in patients— hindered the assessment of artifact-related imaging issues, limiting practical applicability. Another limitation is using burs to create defects. This tends to produce uniform, clear margins, facilitating the detection than it would be with naturally occurring lesions. Acid etching may have provided a more realistic simulation. Additionally, noise was not quantitatively assessed; therefore, any potential influence of voxel-size–related noise on the assessment of dehiscence and fenestrations cannot be ruled out. Moreover, the study evaluated only two voxel sizes and two CBCT software programs, which may limit the generalizability and clinical applicability of the findings.

For future studies, it is recommended to use methods like acid etching to create more irregular defect margins to mimic natural lesions and enhance diagnostic accuracy. Furthermore, incorporating metal restorations into ex-vivo or in-vitro models would account for scattering and beam hardening, and help simulate real clinical conditions. Additionally, future studies should quantitatively assess image noise and systematically evaluate the potential influence of voxel-size–related noise on the detection of dehiscence and fenestrations using objective noise analysis methods. Finally, it is advisable to explore a wider range of voxel sizes and image-analysis software to enhance the generalizability and clinical relevance of the findings.

## Conclusion

Changes in voxel size and software program had little impact on the diagnostic accuracy for detecting fenestrations and dehiscences. CBCT demonstrated good performance in identifying fenestrations but only moderate capability in detecting dehiscences. These results indicate that, although CBCT is a useful imaging tool, its contribution to diagnosing dehiscences is limited by factors such as volume averaging and partial volume effects, and should therefore be interpreted with caution in clinical settings.

## Data Availability

The datasets used during the current study are available from the corresponding author on reasonable request.

## Declarations

This research received an ethics exemption from the Ethics Committee of the Faculty of Dentistry, Ain Shams University, as it does not involve live animals or human participants.
